# Association between Foot Posture Asymmetry and Static Stability in Patients with Knee Osteoarthritis: A Case-Control Study

**DOI:** 10.1155/2020/1890917

**Published:** 2020-06-05

**Authors:** Zehua Chen, Zhen Shen, Xiangling Ye, Jiatao Wu, Huai Wu, Xuemeng Xu

**Affiliations:** ^1^The Fifth Clinical Medical School, Guangzhou University of Chinese Medicine, Guangzhou 510405, China; ^2^Chongqing Traditional Chinese Medicine Hospital, Chongqing 400021, China; ^3^Guangdong Second Traditional Chinese Medicine Hospital, Guangzhou 510405, China

## Abstract

**Background:**

Interlimb asymmetries are considered to be closely related to knee osteoarthritis (KOA), but foot posture asymmetries in patients with KOA are scarcely reported.

**Objectives:**

We aimed to explore the asymmetrical difference in foot posture between the healthy adults and KOA patients and the relationship between foot posture asymmetry and static stability.

**Methods:**

21 subjects suffering from KOA in the patient group (PG) and 21 healthy adults in the control group (CG) were included in this study. Foot postures on both feet were evaluated by using the foot posture index (FPI); subsequently, asymmetrical FPI scores between two feet were calculated for the two groups. Meanwhile, all the participants were tested with a standing on Dynamic and Static Balancing Instrument (Pro-kin 254P, TecnoBody Company, Italy) for bilateral stability assessment, and center of pressure (COP) parameters including sway length (SL, mm) and sway area (SA, mm^2^) were recorded.

**Results:**

Compared to CG, a larger percentage of supinated feet was examined in relatively severe legs (5, 23.81%), relatively moderate legs (4, 19.05%), and merged results (9, 21.43%) of PG (*P* < 0.05), and the percentage of severe asymmetry (38.1%) was higher in the PG (*P* < 0.05). Moreover, these relationships between the absolute value of asymmetry score and SL or SA were significant in CG and PG, the *P* values below 0.01.

**Conclusions:**

Foot posture asymmetry is significantly associated with static stability both in KOA patients and healthy adults, and more severe asymmetry in foot posture was observed in KOA patients, so it is critical to evaluate foot posture asymmetry for treatment and rehabilitation for patients with KOA.

## 1. Introduction

Despite technological advances, knee osteoarthritis (KOA) currently is still a serious public health concern. It is estimated that the prevalence of knee osteoarthritis is up to 21.51% in the middle-aged and elderly, and the most common KOA is the medial type in China [1]. With the growing population of obesity and aging, it will become more and more prevalent, which heavily threatens people's health and constantly increases the economic burden on the family and society. KOA, a multicausation disease, are closely associated with biomechanical changes, in addition to biochemical factors [2]. Furthermore, it is found that changes from the mechanical properties of the adjacent joints can affect the onset of KOA [3]. The ankle and foot play an important role in biomechanical alterations in the lower limbs of individuals with KOA. As the axis of movement in the lower extremity, the ankle is biomechanically related to problems in the knee [4]. An increased knee adduction angle is observed in subjects with early knee osteoarthritis [5], and a lateral shift in foot center of pressure (COP) could reduce the knee adduction moment through shortening the knee lever arm [6]. Thus, postural deformities of the ankle/foot are considered as potential determinants contributing to KOA.

As a simple and reliable method, FPI is widely used to quantify the degree to which a foot is categorized into three as pronated, neutral, and supinated position [7]. Previous studies [8–11] suggested that there were some connections between FPI and KOA. On the one hand, it was reported that some impairments of balance and stability were detected in patients with KOA [12], and FPI was associated with balance and postural stability [13, 14], which was very important to reduce the risk of falling in the elderly [15]. On the other hand, it has been proved that there is a biomechanical link between foot posture and adduction moment/altered frontal knee alignment [8], and supinated FPI is related to pain, function in KOA patients [10]. Moreover, Levinger et al. [16] found that patients with medial compartment KOA exhibited a more pronated foot type than healthy individuals.

In addition, asymmetries including quadriceps strength asymmetry and trunk movement asymmetry were considered to cause the development of KOA [17]. And there was some evidence that foot asymmetry was associated with lower extremity function [18]. Meanwhile, Rokkedal-Lausch et al. [19] indicated that the asymmetrical FPI scores between two feet were not all the same in different subjects. Therefore, asymmetrical foot posture may exist in individuals with KOA accompanied by impairing function.

Recently, rising attention has been paid to the foot posture, and the relevant researches are increasing. Nevertheless, there is no evidence of foot posture asymmetry in KOA patients, and it is still scarcely reported about the differences of foot posture in two feet between patients with KOA and healthy people. Consequently, we aimed to explore the asymmetrical difference in foot posture between healthy people and persons suffering from KOA and the relationship between foot posture asymmetry and posture stability, which would provide references for further clinical practice.

## 2. Methods

### 2.1. Study Design

This research was designed as a case-control study comparing the asymmetrical foot posture in patients with KOA with a control group of healthy adults. It was carried out at the Guangdong Second Traditional Chinese Medicine Hospital from September 15, 2019, to December 24, 2019. Ethical approval was obtained from the Ethics Committee of Guangdong Second Traditional Chinese Medicine Hospital (No. E1923) and registered at the China Clinical Registration Center (Registration No.: ChiCTR1900026067). In this study, all included participants provided written informed consent and could withdraw from the study at any time.

### 2.2. Participants

Patient group (PG) consisted of 25 individuals with KOA diagnosed by the American College of Rheumatology clinical criteria [20]. The other inclusion criteria were (i) age >50 years, (ii) Kellgren/Lawrence [21] (K/L) grade ≥1 in one or both knees, (iii) presence of predominantly medial compartment OA, and (iv) an ability to stand independently on the platform for 30 seconds without any assistive device. The exclusion criteria were (i) presence of any known inflammatory rheumatic disease/arthritis, (ii) concomitant neurologic diseases, such as stroke, Parkinson's disease, and spinal cord injury, (iii) presence of congenital or traumatic lower limb deformity/length discrepancy, and (iv) history of ankle diseases and lower extremity fracture/surgery. In this study, individuals with unilateral or bilateral KOA were included. According to the severity of symptoms evaluated by using VAS (visual analogue scale) motion, the symptomatic leg (or the most symptomatic leg in a case of bilateral involvement) in PG was defined as the relatively serious leg (RSL), the opposite side as the relatively moderate leg (RML).

Control group (CG) consisted of 25 healthy persons. The healthy subjects were included when they met the following inclusion criteria: (i) age >20 years, (ii) no history of neurological or spinal diseases, (iii) no history of ankle diseases and lower extremity trauma/surgery, and (iv) without lower limb length discrepancy/deformity or any other musculoskeletal disorders.

### 2.3. Evaluation of the Foot Posture

Foot Posture Index-6 (FPI-6) [22] was used to evaluate the foot posture on both feet. FPI-6 consists of six items including talar head palpation, curves above and below the lateral malleoli, calcaneal angle, talonavicular bulge, medial longitudinal arch, and forefoot to rearfoot alignment, and each item is scored between -2 and +2. According to the total sum of all items, feet were categorized into three: neutral (0 ≤ FPI ≤ 5), pronated (5 < FPI ≤ 12), and supinated (−12 ≤ FPI < 0). The participants were asked to march on the spot, then settle into a relaxed and comfortable position, standing barefoot on double leg with their arms by the side and looking straight ahead. The participants were required not to swivel during the assessment to avoid affecting the foot posture. All participants were evaluated on both feet. The evaluation was performed by two investigators (ZH.C and H.W), and the intraclass correlation coefficient (ICC) for the interobserver reliability of FPI score was excellent (interrater ICC of 0.923 in PG and interrater ICC of 0.946 in CG).

### 2.4. Evaluation of the Foot Posture Asymmetry

**Figure 1:**
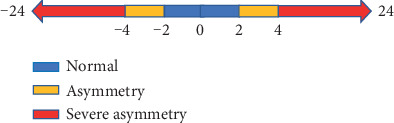
FPI asymmetry score range.

The foot posture asymmetry was assessed with the asymmetry score according to the previous method [19] (difference in FPI score between the two feet), calculated as the FPI score on the right foot minus the FPI score on the left foot. Asymmetry score ranging from -2 to +2 represented normal, −4 ≤ asymmetry score < 2 or +2 < asymmetry score ≤ +4 were asymmetry, and severe asymmetry was <-4 or >4 (shown in Figure 1).

### 2.5. Evaluation of Static Stability

**Figure 2:**
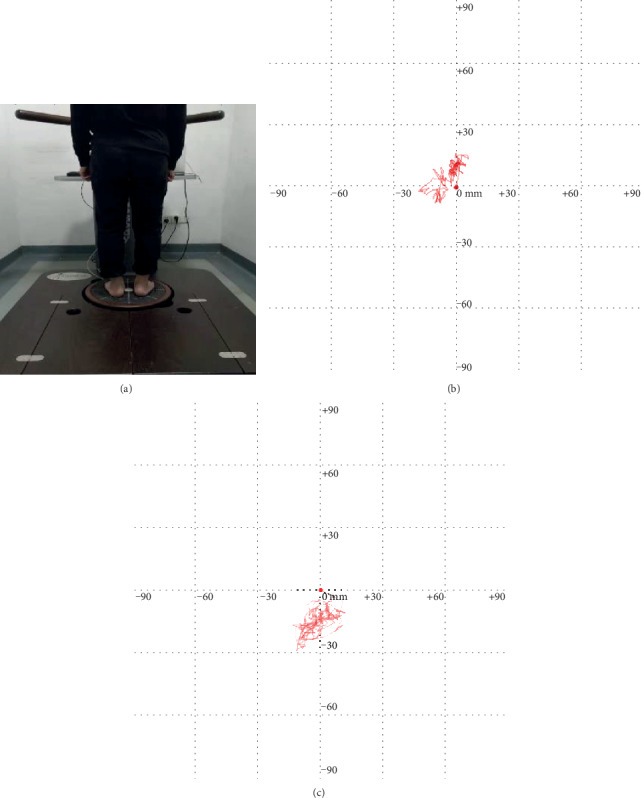
Example of COP sway path test. (a) Participant performing a both-leg static evaluation on the machine; (b) COP sway in a healthy adult; (c) COP sway in a KOA patient.

Center of pressure parameters were determined to assess static balance performance and postural stability during quiet standing [23, 24]. The participants were required to stand statically on a Dynamic and Static Balancing Instrument (Pro-kin 254P, TecnoBody Company, Italy) with two legs for 30 seconds. When they were standing on the platform, the COP sway were documented (Figure 2). During the measurement, the participants were tested with open eyes and their upper limbs placed on the side of body. COP sway length (SL, mm) and sway area (SA, mm^2^) were recorded to evaluate the static balance and stability [25]. The smaller the COP sway, the better the balance ability and the stronger the posture stability.

### 2.6. Statistical Analysis

The sample size (*n* = 21 KOA patients and *n* = 21 controls) was calculated to yield an 85% power, and *α* = 0.05. Analyses were conducted with the use of SPSS25.0 statistical software. Continuous characteristics of the study were checked for normality using the Shapiro-Wilk test. *T* test or nonparametric test (Mann–Whitney) was used to assess the differences between two groups. The categorical variables were assessed by chi-squared test for between-group comparison. Spearman test was conducted for correlation analysis between asymmetrical scores and COP sways. All continuous variables were presented as mean ± standard deviations. Statistical significance was accepted at *P* < 0.05.

## 3. Results

### 3.1. Participants Characteristics

Two groups participated in the study: 21 patients with medial KOA were included in the PG and 21 healthy adults were included in the CG according to the inclusion and exclusion criteria. Baseline demographics of both groups were summarized in Table 1. The age, weight, height, and BMI of the two groups were closely similar, but the gender analysis showed significant difference between both groups because KOA was more common in females, and subsequently, more females were involved. In PG, of the most symptomatic knees evaluated, according to the K/L radiologic severity of KOA, 0 (0%), 1(4.8%), 7 (33.3%), and 13 (61.9%) were graded as K/L grade 1, 2, 3, and 4 (3.210.42), respectively.

### 3.2. Foot Posture Analysis

**Figure 3:**
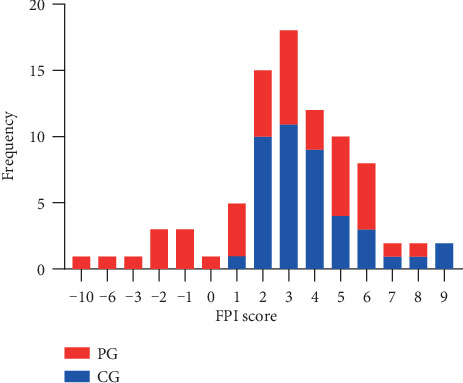
The numbers of feet with various FPI score in PG and CG. PG: patient group, CG: control group.

**Figure 4:**
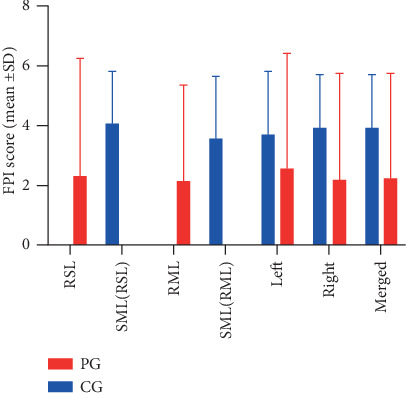
Comparison of FPI score between PG and CG. PG: patient group, CG: control group; SD: standard deviation; RSL: relatively severe leg; RML: relatively moderate leg; SML(RSL): side-matched leg to relatively severe leg; SML(RML): side-matched leg to relatively moderate leg; ^∗^*P* < 0.05.

As was illustrated in Figure 3, FPI mainly centered on 0 to 5 in CG, which revealed a neutral foot was common in healthy subjects, whereas there were some negative values of FPI representing the supinated feet in the PG. When FPI total score was compared, the results did not differ statistically between the PG and CG (Figure 4). Of note, the RSL and RML were defined on the basement of symptom severity of KOA. Thus, both RSL and RML may be the leg on the right or left side. For this study, the RSL consisted of 10 left legs and 11 right legs, whereas the RML included 11 left legs and 10 right legs. Therefore, side-matched legs (SML) were selected from CG by using a balanced randomization method and used to compare with the corresponding RSL and RML of PG, respectively. RSL (23.81%), RML (19.05%), and merged results (21.43%) in PG showed a more supinated foot posture than SML(RSL) (0), SML(RML) (0), and the merged results (0) in CG, respectively. Legs with normal foot posture were significantly less in PG in contrast to CG (*P* < 0.05). The corresponding data were shown in Table 2.

### 3.3. Foot Posture Asymmetry Analysis

**Figure 5:**
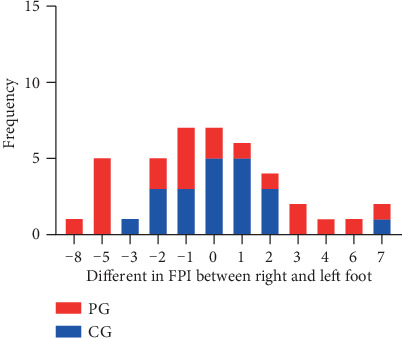
The numbers of subjects with various FPI asymmetry score in PG and CG. PG: patient group; CG: control group.

Asymmetry score, difference in FPI between the right foot and left foot, was calculated for each participant from both groups, and their asymmetry score distributions were shown in Figure 5. It was demonstrated that the asymmetry score mainly ranged from -2 to 2 in the CG, whereas it was distributed widely in PG. After the asymmetry score was categorized into three types, normal, asymmetry, and severe asymmetry, it was detected that the percentage of severe asymmetry was higher in the PG (38.1%) than the CG (4.8%), and the ratio of normal was lower in the PG (47.62%) than in the CG (90.48%) (Table 3).

### 3.4. COP Sways Analysis

With regard to COP sway, a greater SL was observed in the PG (555.52 ± 177.95) than in the CG (352.38 ± 77.72), and SA was significantly larger in the PG (1061.28 ± 639.49) than in the CG (335.00 ± 201.48), *P* < 0.01 and *P* < 0.01, respectively (Table 4).

### 3.5. Association between FPI Scores and COP Sways

The results of multiple regression analyses exhibiting the association between FPI and COP sway are shown in Table 5. Both in the PG and the CG, an increased FPI (i.e., more pronated foot) on the both feet was significantly associated with increases in SL (*P* < 0.05) and SA (*P* < 0.05), except that the association between FPI on the right foot in the CG and SA did not show significant difference (*P* = 0.56). Meanwhile, these relationships between the absolute value of asymmetry score and SL or SA were still significant in CG [(73.36 per degree, 95% CI: 46.49, 100.22; *R*^2^ = 0.27; *P* < 0.01) and (66.75 per degree, 95% CI: 20.15, 110.34; *R*^2^ = 0.10; *P* < 0.01), respectively] and PG [(113.14 per degree, 95% CI: 92.25, 134.03; *R*^2^ = 0.59; *P* < 0.01) and (826.23 per degree, 95% CI: 703.59, 948.87; *R*^2^ = 0.69; *P* < 0.01), respectively].

## 4. Discussion

FPI, a quick, easy, and cost-effective method, is widely used to comprehensively evaluate foot position [26]. Compared to the electromagnetic track system, it could predict 80% of the variance [22]. Several studies [27–29] reported the excellent reliability of FPI, and it also demonstrated a good inter- and intrarater agreement when used by the inexperienced rater [28, 29]. In the present study, the excellent FPI intrarater reliability of 0.988 was also observed in KOA patients. As was previously reported, foot posture was closely related to age, and a more pronated foot posture was found in subjects with KOA [9, 16]. Moreover, the results derived from this study showed that, for KOA patients, the number of pronated feet was significantly larger in RHS, and both pronated feet and supinated feet were significantly more in RLS, and pronated and supinated foot postures in people suffering from KOA are more than those in healthy adults. However, we found no significant difference in the comparison of FPI scores between the two groups, which was also not the same as the results reported by Abourazzak et al. [9] and Levinger et al. [16]. On the one hand, according to the FPI, negative values mean supinated foot posture, and positive values mean pronated foot posture, and KOA was proved to be associated with supinated FPI according to the K/L radiologic severity of KOA [10]. Thus, it might be the reason for the different results that included patients differed in severity of KOA between the studies and more KOA patients with supinated foot in the present study. Due to the special distribution of the values, no difference in FPI scores was detected. On the other hand, there is a significantly biomechanical connection between frontal plane alignment and foot posture in patients with KOA [8, 11]. Abnormal foot posture, including pronated and supinated foot posture, through affecting the moment arm and reaction force, exerts an impact on the force distribution inside the knee joint [30]. To some degree, it is also the reason why there are more abnormal foot postures in KOA patients.

To the best of our knowledge, this is the first time to evaluate the foot posture asymmetry in KOA patients and compare their foot posture asymmetries to the healthy adults. It was reported that there were some asymmetries of two legs in KOA patients [17, 18], and they were related to an increased risk of injury and exerted impacts on lower extremity function [18]. The results of this study revealed that individuals with KOA revealed a higher percentage of severe asymmetry. It could be explained that biomechanical alterations in the affected leg would cause compensatory changes in the contralateral side, correspondingly, changes of foot posture emerged, and even led to the onset of KOA in the asymptomatic legs; ultimately, the difference of foot posture in both legs might increase. Creaby et al. [31] demonstrated that, for KOA patients, the presence of unilateral pain was associated with asymmetry in knee biomechanics, whereas bilateral pain is related to symmetry. Therefore, a greater difference of FPI score between both legs detected in KOA patients, especially in patients with unilateral KOA, could be due to the influence of the affected limb on the opposite side.

The other main finding of the present study was the relationship between foot posture and COP sway. Cote et al. [32] suggested that postural stability was mainly affected by foot posture. Differences in foot posture made differences in the postural control, for example, postural sway increased by pronation foot [33]. In the current study, we observed that patients with KOA had significantly larger COP sways than healthy adults. And an increasing COP sway (SL and SA) was significant associated with increases in FPI (i.e., pronation direction) on the left or right foot, which were detected both in CG and PG. Most importantly, our findings demonstrated a significant association between asymmetrical FPI scores and static stability; moreover, the connections were observed both in KOA patients and healthy adults. It could be explained by the reasons that foot posture asymmetry would alter the body center of gravity and consequently result in balance impairment.

In a word, foot posture alterations are closely related to KOA. Significantly, worse postural stability is examined in patients with KOA than in health subjects, and it is related to their foot posture asymmetry. Wedge insole can effectively correct the foot posture in the affected leg and modulate the COP, which is beneficial to prevent the development of KOA by reducing the adduction moment [34, 35]. Due to the variable foot posture in subjects with KOA, lateral wedge insoles should not always be recommended, and it is critical to select a suitable one. However, of note, to address foot posture asymmetry, we should also pay attention to the unaffected side.

There are some limitations in the present study: firstly, due to the higher incidence of KOA in old women than in men, the gender of the included KOA patients was not equal; secondly, the K/L radiologic severities of KOA patients included in this study were mainly graded as 3 and 4, and people with severe KOA were prone to have more serious deformities and dysfunctions, which could be the reason that the percentage of severe asymmetry foot postures in the included KOA patients is significantly larger than the health adults, whereas the percentage of the pronated foot and asymmetry showed no significant difference; thirdly, the foot posture was not distributed normally, and supinated foot was not examined in the included healthy adults, because of little percentage of supinated foot in the healthy adults.

## 5. Conclusion

This study confirms that KOA patients and healthy adults display different characteristics of foot posture asymmetry and COP sway. Moreover, foot posture asymmetry is significantly associated with static stability both in KOA patients and healthy adults, and more severe asymmetry in foot posture is examined in KOA patients. After evaluated foot posture, it will be helpful to improve the balance and stability and reduce the symptoms for the KOA patients by correcting asymmetry foot posture. Therefore, the foot posture asymmetry should be taken into consideration during the treatment and rehabilitation for patients with KOA.

## Figures and Tables

**Table 1:** Baseline characteristics of the study volunteers.

	PG (*n* = 25)	CG (*n* = 25)	*P* values
Age (years)	61.52 ± 7.47	50.52 ± 18.40	0.12
Female/male	19/2	9/12	< 0.01
Height (cm)	160.38 ± 6.89	164.38 ± 6.99	0.07
Weight (kg)	63.76 ± 8.02	60.14 ± 11.86	0.25
Body mass index (kg/m^2^)	24.75 ± 2.26	22.12 ± 3.35	0.07
K/L grade	3.21 ± 0.42	—	—

PG: patient group; CG: control group; K/L: Kellgren/Lawrence.

**Table 2:** Comparison of foot posture between the two groups.

Groups		Foot posture		
		Supinated	Neutral	Pronated
PG	RSL	5 (23.81%) ^∗^	13 (61.90%)	3 (14.29%)
	RML	4 (19.05%) ^∗^	13 (61.90%)	4 (19.05%)
	Merged	9 (21.43%) ^∗^	26 (61.90%) ^∗^	7 (16.67%)
CG	SML(RSL)	0	17 (80.95%)	4 (19.05%)
	SML(RML)	0	18 (85.71%)	3 (14.29%)
	Merged	0	35 (83.33%)	7 (16.67%)

PG: patient group, CG: control group; RSL: relatively severe leg; RML: relatively moderate leg; SML(RSL): side-matched leg to relatively severe leg; SML(RML): side-matched leg to relatively moderate leg; ^∗^*P* < 0.05.

**Table 3:** Comparison of foot posture asymmetry between the two groups.

Groups	Foot posture asymmetry		
	Normal	Asymmetry	Severe asymmetry
PG	10 (47.62%) ^∗^	3 (14.29%)	8 (38.10%) ^∗^
CG	19 (90.48%)	1 (4.76%)	1 (4.76%)

PG: patient group, CG: control group; ^∗^Compared to CG, *P* < 0.05.

**Table 4:** Comparison of COP sway between the two groups.

Groups	COP sways	
	SL (mm)	SA (mm^2^)
PG	555.52 ± 177.95^∗∗^	1061.28 ± 639.49^∗∗^
CG	352.38 ± 77.72	335.00 ± 201.48

PG: patient group, CG: control group; ^∗∗^Compared to CG, *P* < 0.01.

**Table 5:** The association of FPI scores with COP sways.

Dependent variables	Beta (95% CI) of COP sways, per degree	
	SL (mm)	SA (mm^2^)
CG		
Left	37.35 (20.61, 54.09) ^∗∗^	28.10 (1.44, 54.76) ^∗^
Right	36.39 (4.04, 68.75) ^∗^	14.29 (-34.54, 63.11)
Asymmetrical scores	73.36 (46.49, 100.22) ^∗∗^	66.75 (20.15, 110.34) ^∗∗^
PG		
Left	662.56 (633.37, 691.76) ^∗∗^	662.56 (633.37, 691.76) ^∗∗^
Right	49.44 (45.90, 52.98) ^∗∗^	158.62 (89.86, 227.87) ^∗∗^
RSL	34.51 (22.10, 46.91) ^∗∗^	437.64 (418.07, 457.21) ^∗∗^
RML	56.96 (51.89, 62.03) ^∗∗^	93.76 (5.02, 182.50) ^∗^
Asymmetrical scores	113.14 (92.25, 134.03) ^∗∗^	826.23 (703.59, 948.87) ^∗∗^

95% CI: 95% confidence interval; COP: center of pressure; PG: patient group; CG: control group; NE: not estimable; RHS: relatively severe leg; RML: relatively moderate leg; ^∗^*P* < 0.05; ^∗∗^*P* < 0.01.

## Data Availability

The data used to support the findings of this study are available from the corresponding author upon request.
